# Pharmacology-Based Prediction of the Targets and Mechanisms for Icariin against Myocardial Infarction

**DOI:** 10.3390/medicina59030420

**Published:** 2023-02-21

**Authors:** Zunping Ke, Yuling Wang, Guzailinur Silimu, Zhangsheng Wang, Aimei Gao

**Affiliations:** 1 Department of Geriatrics, Shanghai Fifth People’s Hospital, Fudan University, Shanghai 200240, China; 2 Department of Gastroenterology, Changhai Hospital, Naval Military Medical University, No. 168 Changhai Road, Shanghai 200433, China; 3 Department of Cardiology, People’s Hospital of Zepu County, Xinjiang 844899, China; 4 Department of Cardiology, Shanghai Fifth People’s Hospital, Fudan University, Kashgar 200437, China; 5 Department of Clinical Pharmacy, Shanghai General Hospital, Shanghai Jiao Tong University School of Medicine, Shanghai 200080, China

**Keywords:** computational network pharmacology, molecular docking, myocardial infarction, Icariin

## Abstract

*Background and Objectives:* This study aims to illustrate the mechanisms underlying the therapeutic effect of Icariin after myocardial infarction (MI). *Materials and Methods:* Based on the network pharmacology strategy, we predict the therapeutic targets of Icariin against MI and investigate the pharmacological molecular mechanisms. A topological network was created. Biological process and Kyoto Encyclopedia of Genes and Genomes pathway enrichment were also performed. We also conducted the molecular docking analysis to stimulate the component–target interaction further and validate the direct bind effect. *Results:* Network pharmacology analysis identified 61 candidate genes related to the therapeutic effect of Icariin against MI. *EGFR*, *AKT1*, *TP53*, *JUN*, *ESR1*, *PTGS2*, *TNF*, *RELA*, *HSP90AA1*, *and BCL2L1* were identified as hub genes. The biological processes of the candidate targets were significantly involved in the reactive oxygen species metabolic process, response to hypoxia, response to decreased oxygen levels, response to oxidative stress, regulation of reactive oxygen species metabolic process, and so forth. Overall, biological process enrichment analysis indicated that the protective effect of Icariin against MI might be associated with oxidative stress. Moreover, the pathway analysis showed that the candidate targets were closely associated with lipid and atherosclerosis, AGE-RAGE signaling pathway in diabetic complications, HIF-1 signaling pathway, etc. We identified the conformation with the lowest affinity score as the docking conformation. The simulated molecular docking was displayed to illustrate the topical details of the binding sites between Icariin and TNF protein. *Conclusions:* This study provides an overview of the mechanisms underlying the protective effect of Icariin against MI.

## 1. Introduction

Myocardial infarction (MI) is a common ischemia heart disease caused by the obstructed arterial inflow to the heart and subsequent myocardium impairment [1,2]. Currently, MI is one of the most important risk factors for heart failure (HF), and it has greatly reduced the population life expectancy across the globe [3]. Over the last decade, MI has become one of the leading causes of death worldwide [4]. In the US, it is estimated that the annual incidence of MI is approximately 605,000 new cases and 200,000 recurrent cases a year [5]. The primary pathological processes leading to MI include artery inflammation, lipid deposition, and metabolic disorders caused by many risk factors [6]. Reactive oxygen species (ROS) have been well-demonstrated in aggravating the adverse effects of myocardial infarction and many other diseases [7,8,9,10]. Despite the advances in the cellular and molecular mechanisms underlying myocardial ischemia/reperfusion injury, treating ischemic heart disease remains challenging.

Epimedii Herba, also referred to as Horny Goat Weed, is a traditional Chinese herbal medicine [11]. Epimedii Herba has shown various health benefits, including therapeutic potential on cardiovascular diseases [11]. Icariin is the major bioactive pharmaceutical component isolated from Epimedii Herba. Many potential mechanisms underlying the cardioprotective effects of Icariin have been revealed, including inhibiting oxidative stress, attenuating DNA damage, correcting endothelial dysfunction, preventing platelet activation, and so forth [12,13]. Importantly, Icariin can inhibit oxidative stress injury, prevent mitochondrial oxidative damage, and reduce cardiomyocyte apoptosis [1,2]. These characteristics could potentially protect the heart cell from the damage caused by free radicals and improve the blood flow. Although some pharmacological studies reported the beneficial effect of Icariin in preventing cardiac ischemia/reperfusion injury [14], improving cardiac remodeling [15], and inhibiting ferroptosis of cardiomyocytes [16], it remains unclear whether Icariin could provide clinical benefits for MI patients. The computational network pharmacology-based investigation on the potential effect of MI could provide more preliminary evidence for Icariin administration.

Network pharmacology is an integrative computational method that integrates systematic medicine with information science and establishes a protein–compound and disease–gene network [17]. Combining the network analysis and computational methods, network pharmacology can be used to understand the complex interactions between drugs, targets, and biological pathways. The goal of network pharmacology is to identify the most effective combinations of drugs for treating various diseases by analyzing the complex interactions between drug targets and biological pathways. To comprehensively understand of the interaction between drugs and diseases in the human body, multiple disciplines were involved in network pharmacology, including biology, chemistry, computer science, and mathematics. Network pharmacology can also be used to develop new drugs, improve existing treatments, better understand the underlying mechanisms of disease, and re-purpose approved drugs, which provides an opportunity to systematically extend the druggable space of proteins implicated in various diseases [18,19]. The analysis based on network pharmacology usually follows these steps: identify drug compounds and disease-related target genes, construct a protein–protein interaction network based on the identified genes, and visualize the gene network [17]. To date, network pharmacology has been widely used for the unbiased elucidation of drug mechanisms and systematic prediction of effective therapeutic combinations [20,21]. This study used network pharmacology methods to investigate the mechanisms of Icariin on MI.

## 2. Materials and Methods

### 2.1. Data Sources and Targets Fishing

PubChem (https://pubchem.ncbi.nlm.nih.gov/, accessed on 14 October 2022) is a free online database of chemical information created by the National Institutes of Health. It provides a repository of over 39 million chemical compounds with associated data such as chemical structures, 3D models, bioactivities, reactions, and spectra. Compounds can be searched and visualized using the PubChem Structure Search and PubChem Compound Search tools, and data can be downloaded in several different file formats. PubChem also provides access to related resources, such as databases of potential drug targets, gene expression data, and toxicity profiles. We acquire Icariin’s 2D Structure and Canonical SMILES (Simplified Molecular Input Line Entry System) from the PubChem database using the keyword “Icariin”. Canonical SMILES is a molecular representation format used to describe the structure of a chemical compound. It is a text-based representation of a molecular structure, using a string of characters to represent the bonds and atoms in a molecule. It is a standardized system which is used to uniquely identify molecules and aid in searching databases.

The predictive targets of icariin were harvested from the Swiss Target Prediction (www.swisstargetprediction.ch, accessed on 15 October 2022) [22] and STITCH (stitch.embl.de, accessed on 15 October 2022) [23] databases. Swiss Target Prediction is a database that contains predicted targets for small molecules [22]. It is based on a machine learning approach and is designed to provide an easily accessible source for target prediction of small molecules. The database provides a comprehensive overview of the predicted targets for a given small molecule, which can be used to inform preclinical drug development. The Swiss Target Prediction database contains over 1.5 million predicted targets for small molecules and is updated regularly with new compounds and targets. STITCH is a database that provides access to interactions between chemical compounds and proteins [23]. It is a comprehensive resource of over 29 million interactions between small molecules and proteins, which can be used to generate hypotheses about compound–protein associations and potential drug targets. The database also contains information about protein–protein interactions, protein families, pathways, and protein post-translational modifications.

Moreover, target genes related to MI were collected with the keyword “myocardial infarction” in TTD (db.idrblab.net/ttd, accessed on 15 October 2022) [24], DrugBank, DisGeNET (www.disgenet.org, accessed on 15 October 2022) [25], and CTD database (ctdbase.org, accessed on 15 October 2022) [26] databases, which contain genes and variants associated with human diseases. The TTD is an online resource created to provide comprehensive and up-to-date information about therapeutic targets and their associated drugs [24]. It contains information on over 7500 drug targets and more than 7000 drug products, including drugs in clinical and pre-clinical development, as well as approved drugs. The TTD includes detailed information about the interaction between drugs and target molecules, including the type of interaction, mechanism of action, and the effects of the drug–target interaction. The database also includes information about drug metabolism, toxicity and other pharmacological data. DrugBank Database is a comprehensive, publicly accessible, online drug information resource that contains detailed information on thousands of drug molecules. It includes detailed information on small molecules, biologics, and drug-to-drug interactions. DisGeNET is an open access database that provides comprehensive information on human diseases and their associated genes [25]. It is a comprehensive and integrated resource that provides access to a large collection of data related to the genetic factors involved in the etiology of human diseases. It is an open-access database that contains data from more than 200 sources, including both curated and automatically generated information. DisGeNET also provides access to a variety of tools to help researchers identify and analyze disease-related genes. The Comparative Toxicogenomics Database is a web-based, open-access resource created to help researchers better understand the complex relationships between chemicals, genetic variants, and diseases [26]. It provides detailed information on gene–chemical, gene–gene, gene–disease, and other biological interactions. CTDbase.org also provides access to curated biological pathways, pathway-based analysis tools, and chemical–disease associations. This resource is designed to facilitate the integration of toxicogenomics and environmental health research.

After acquiring target genes, we used the UniProt (www.uniprot.org, accessed on 15 October 2022) [27] database to match gene symbols with UniProt ID, and we removed duplicated genes and standardized all the results. Only the genes from “Homo sapiens” were selected for the following analyses. Finally, we overlapped the Icariin targets and MI-related genes to acquire candidate therapeutic targets of Icariin against MI.

### 2.2. Construction of Protein–Protein Interaction Network and Topological Analysis

STRING (http://string-db.org, accessed on 15 October 2022) [28] is a protein–protein interaction database, which aims to collect, score, and integrate all publicly available sources of protein–protein interaction (PPI) information. It contains information from diverse sources, including experimental databases, computational prediction methods, and interactions extracted from the literature [28]. The database allows researchers to explore the protein–protein interactions of a particular gene or protein and to visualize the network of interactions. This can facilitate research in network pharmacology by providing researchers with a comprehensive view of a drug’s target proteins and their interactions, which can help in predicting its mechanism of action, identifying potential off-target effects, and elucidating the pharmacological pathways involved. STRING database has provided the researchers with a useful tool to create PPI networks, which can be used to draw insights into the pharmacological pathways involved and to identify potential drug effects [28]. We input the candidate genes into the STRING database and set the organism as Homo sapiens. Then, the STRING database matched the candidate genes with proteins automatically. It should be noted that one gene would be only matched to one protein in the STRING database. We used the STRING database to collect possible interactions of target proteins with a medium confidence score of >0.4. Both functional and physical protein associations were analyzed. Resultant PPI data were visualized by Cytoscape software (v3.7.2) [29].

An analyzer (a plug-in of Cytoscape) was utilized in analyzing topological parameters of mean and maximum degrees of freedom in the PPI network. Further, based on mixed character calculation, we identified the top 10 hub genes using the Cytoscape plug-in software “cytoHubba”.

### 2.3. Biological Process and Pathway Enrichment Analysis

To reveal the biological function of the target genes, we performed Gene Ontology (GO) and Kyoto Encyclopedia of Genes and Genomes (KEGG) pathway enrichment analysis. GO is a controlled vocabulary of biological terms used to describe gene products in any organism. The GO vocabulary is used to classify genes and gene products into three distinct categories: molecular function, biological process, and cellular component. GO is used to aid in the interpretation of gene expression data, to provide insight into the underlying molecular mechanisms of a given phenotype, and to identify network and pathway relationships between genes and gene products. The KEGG pathway database is an online resource that provides comprehensive information on molecular-level pathways, networks and interactions in various organisms, including humans. It contains manually curated information on metabolic, genetic, and regulatory networks, as well as on the evolution and development of biological systems. The information is organized into a series of pathways and maps that connect genes and proteins to their metabolic, regulatory, and signaling functions. KEGG pathways are used to analyze and visualize large-scale omics data, and to infer underlying biological networks and functions. The enriched biological processes and KEGG signaling pathways related to the candidate targets of Icariin against MI were visualized. We used default parameters in the study unless otherwise specified.

### 2.4. Component–Target Molecular Docking

Computational molecular docking is a computational technique used to predict the binding affinity of a given molecule to a target protein or ligand [30]. Since it can provide insight into the binding mechanisms of drug–target interactions, computational molecular docking has become a vital method to explore the component–target interaction and predict the binding model [30]. It is a powerful tool for understanding the structure–activity relationships of molecules and helps in the design of new molecules with desired binding properties. Component–target molecular docking involves the use of a scoring function to evaluate the binding affinity of a given molecule to its target. This scoring function is a mathematical representation of the interaction energy between the molecule and target. The molecule is placed at various orientations relative to the target, and the energy of the interaction is evaluated for each orientation. The orientation that yields the lowest energy is chosen as the optimal binding conformation. The process of component–target molecular docking typically involves two steps. First, the receptor–ligand complex is modelled using a physical model. This model is used to generate the coordinates of the ligand and the receptor molecule. Second, the ligand is docked into the receptor molecule using a scoring function. This scoring function is a combination of the energy of the receptor–ligand complex and the entropic contribution due to the internal motions of the ligand molecule.

We performed the molecular docking analysis to stimulate the component–target interaction further and validate the direct bind effect. We obtained the three-dimensional structure of the potential hub gene (treated as a receptor) from the RCSB PDB database (https://www.rcsb.org/, accessed on 15 October 2022). The AutoDock Tool software was applied to pre-process the protein structure by removing the water molecules and adding the nonpolar hydrogen atoms. The 2D structure of Icariin was obtained from PubChem, transformed by the Open Babel software, and saved in the PDBQT format as a ligand via the AutoDock software. Next, we used the Autodock Vina software to predict the molecular docking site, and the affinity score (binding energy) of each conformation pair was calculated. The lower affinity score indicates a stronger bond. We identified the conformation with the lowest affinity score as the docking conformation. Finally, the conformation between Icariin and the potential target was visualized by PyMOL software.

## 3. Results

### 3.1. Candidate Targets of Icariin against MI

**Figure 1:**
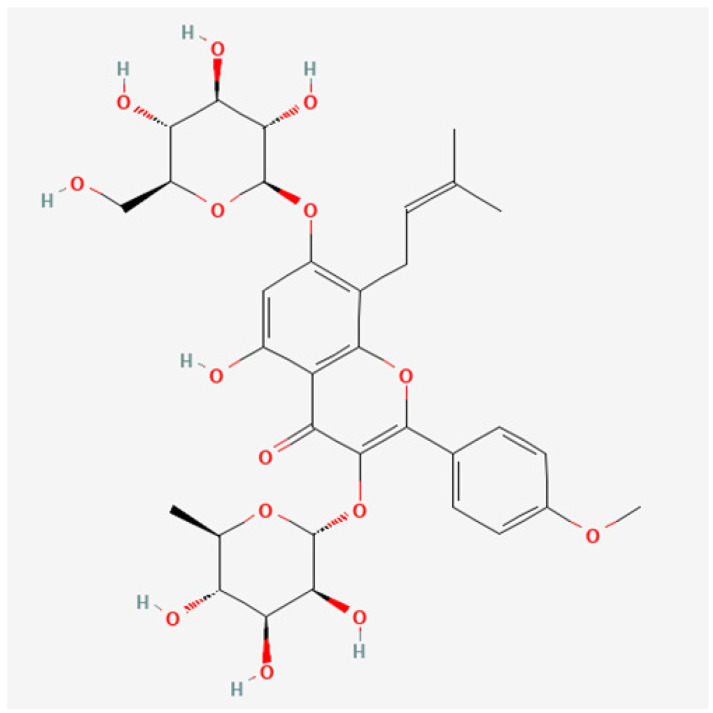
The 2D structure of Icariin. We acquire Icariin’s 2D Structure and Canonical SMILES (Simplified Molecular Input Line Entry System) from the PubChem database using the keyword “Icariin.” The PubChem CID is 5318997, and the canonical SMILES of Icariin is CC1C(C(C(C(O1)OC2=C(OC3=C(C2=O)C(=CC(=C3CC=C(C)C)OC4C(C(C(C(O4)CO)O)O)O)O)C5=CC=C(C=C5)OC)O)O)O.

The canonical SMILES of Icariin is CC1C(C(C(C(O1)OC2=C(OC3=C(C2=O)C(=CC(=C3CC=C(C)C)OC4C(C(C(C(O4)CO)O)O)O)O)C5=CC=C(C=C5)OC)O)O)O. The 2D Structure of Icariin is illustrated in Figure 1. The PubChem CID is 5318997.

**Figure 2:**
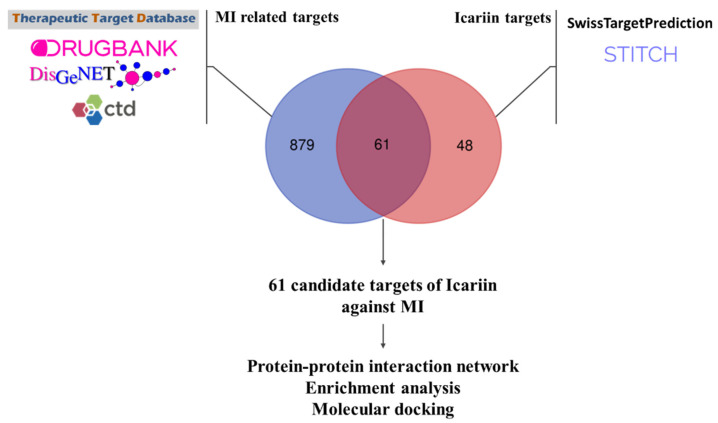
Target identification of Icariin to treat myocardial infarction. After standardization and duplicate removal, 940 MI-related targets and 109 Icariin targets were obtained. We overlapped MI-related genes and Icariin targets, and 61 candidate drug–disease inter-action genes were acquired. The candidate drug–disease interaction genes included *PRKCE, XDH, ADRA2C, NOS2, PRKCZ, SLCO1B3, ALOX5, CYP1A2, BACE1, PRKCB, SLCO2B1, EGFR, IKBKB, ADORA1, ALDH2, IL2, BCHE, BCL2, OPRD1, ADRA2A, PDE5A, F7, KCNH2, F10, LDHB, CYP1A1, BCL2L1, TNNI3, ADORA3, HSP90AB1, RELA, PRKCD, NOS3, SLC29A1, ABCB1, PLG, SER-PINE1, CYP19A1, TP53, TNF, DRD2, LDHA, AKT1, PRKACA, F2, PTGS2, SQLE, KLK1, NFKB1, ABCC1, NOX4, BAD, ESR1, SCARB1, JUN, ACHE, PRKCA, CYP1B1, APP, RPS6KA3, HSP90AA1*.

After standardization and duplicate removal, 940 MI-related targets and 109 Icariin targets were obtained. The detailed MI-related genes and Icariin target genes are provided in Supplement Tables S1 and S2, respectively. We overlapped MI-related genes and Icariin targets, and 61 candidate drug–disease interaction genes were acquired (Figure 2). The candidate drug–disease interaction genes included *PRKCE, XDH, ADRA2C, NOS2, PRKCZ, SLCO1B3, ALOX5, CYP1A2, BACE1, PRKCB, SLCO2B1, EGFR, IKBKB, ADORA1, ALDH2, IL2, BCHE, BCL2, OPRD1, ADRA2A, PDE5A, F7, KCNH2, F10, LDHB, CYP1A1, BCL2L1, TNNI3, ADORA3, HSP90AB1, RELA, PRKCD, NOS3, SLC29A1, ABCB1, PLG, SERPINE1, CYP19A1, TP53, TNF, DRD2, LDHA, AKT1, PRKACA, F2, PTGS2, SQLE, KLK1, NFKB1, ABCC1, NOX4, BAD, ESR1, SCARB1, JUN, ACHE, PRKCA, CYP1B1, APP, RPS6KA3, HSP90AA1*.

### 3.2. Network and Topological Analysis

**Figure 3:**
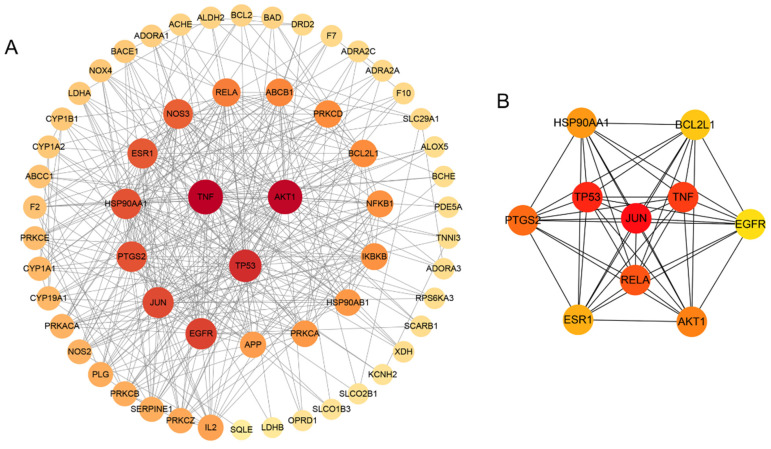
Network and topological analysis on the candidate targets. (**A**) PPI network of Icariin against myocardial infarction. The size of nodes and the color intensity from dark to light were proportional to the degree of centrality by topology analysis. The darker the color, the more critical the node. (**B**) Top 10 Hub targets of Icariin against myocardial infarction, including *EGFR, AKT1, TP53, JUN, ESR1, PTGS2, TNF, RELA, HSP90AA1, BCL2L1*.

We imported the 61 potential targets into the STRING database and obtained the PPI network, which contained 61 nodes and 362 edges with an average node degree of 12.1 (Figure 3A). The cytoHubber revealed the top 10 hub genes ranked by maximal clique centrality (MCC) according to the topological parameters of the interaction network: *EGFR*, *AKT1*, *TP53*, *JUN*, *ESR1*, *PTGS2*, *TNF*, *RELA*, *HSP90AA1*, *BCL2L1* (Figure 3B).

### 3.3. Biological Process and KEGG Pathway Enrichment Analysis

**Figure 4:**
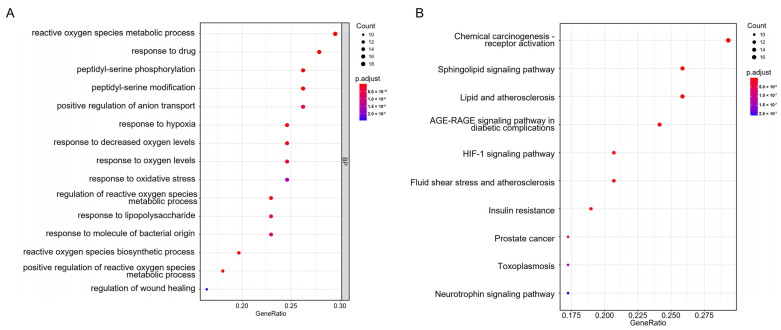
Biological process and pathway enrichment analyses for Icariin against myocardial infarction. (**A**) The enriched primary biological processes, including the reactive oxygen species metabolic process, response to hypoxia, response to decreased oxygen levels, response to oxidative stress, regulation of reactive oxygen species metabolic process, and so forth. (**B**) The enriched KEGG pathways, including lipid and atherosclerosis, AGE-RAGE signaling pathway in diabetic complications, HIF-1 signaling pathway, and so forth.

Biological process and KEGG pathway enrichment analyses were performed to clarify the characteristics of Icariin-related targets. As illustrated in Figure 4A, the biological processes of the candidate targets were significantly involved in the reactive oxygen species metabolic process, response to hypoxia, response to decreased oxygen levels, response to oxidative stress, regulation of reactive oxygen species metabolic process, and so forth. Overall, biological process enrichment analysis indicated that the protective effect of Icariin against MI might be associated with oxidative stress.

Moreover, the KEGG pathway analysis showed that the candidate targets were closely associated with lipid and atherosclerosis, AGE-RAGE signaling pathway in diabetic complications, HIF-1 signaling pathway, etc. (Figure 4B).

### 3.4. Molecular Docking

**Figure 5:**
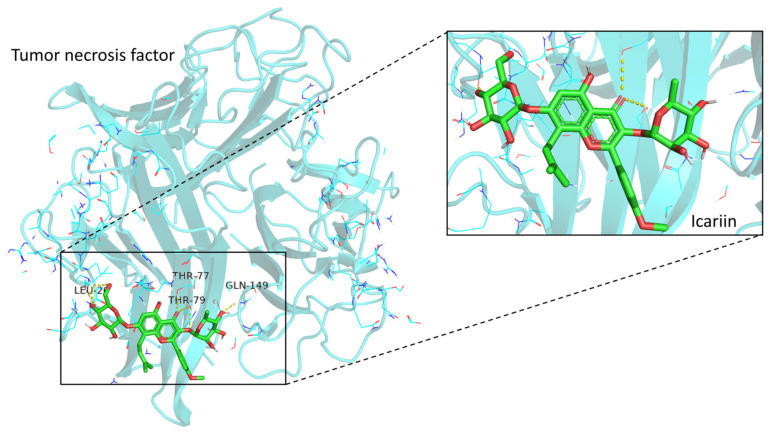
Molecular docking between Icariin and TNF protein. The enlarged view on the right showed the interactions of ligands and amino acid residues. Three hydrogen bonds were formed: Icariin UNL1:H—TNF A:PRO117:O, Icariin UNL1:O—TNF C:TYR119:HN, and Icariin UNL1:O—TNF C:TYR110:HH.

Based on the integration of the results from the PPI network, we selected the TNF as the potential target in molecular docking. The structure of the human protein encoded by the *TNF* gene was obtained from the RCSB Protein Data Bank (PDB DOI: 10.2210/pdb1TNF/pdb). After pre-processing, we performed molecular docking between Icariin and TNF protein. Conformation with high binding energy (affinity score below −5 kcal/mol) was considered a stable structure. We identified the conformation with the lowest affinity score as the docking conformation. The simulated molecular docking is displayed in Figure 5 to illustrate the topical details of the binding sites between Icariin and TNF protein.

## 4. Discussion

MI is a clinical event characterized by the acute reduction in or cessation of blood flow to a portion of the heart muscle, leading to ischemic injury and cell death. It is typically caused by the occlusion of a coronary artery by a thrombus or embolus, which is usually formed on top of a ruptured vulnerable plaque. The incidence of MI has been declining, but it remains a leading cause of death and disability worldwide [5,31]. The risk factors for MI include advanced age, male gender, smoking, hypertension, dyslipidemia, diabetes, family history, sedentary lifestyle, and so forth [5,31]. Although the advances in diagnosis and treatment strategies have reduced the mortality caused by MI, most MI patients still suffer from an irreversible pathological evolution, which might eventually result in left ventricle dysfunction or heart failure [32]. The exploration of drugs against MI is a hot spot of cardiovascular research.

Epimedii Herba is a perennial herb native to China and other parts of Asia [11]. It belongs to the Berberidaceae family and has been used for centuries in traditional Chinese medicine for various purposes. The potential cardiovascular benefits of Epimedii Herba include reducing blood pressure, improving blood circulation, and reducing the risk of heart disease. Epimedii Herba includes multiple active compounds, such as include flavonoids, icariin, and prenylflavonoids [33]. Among the pharmacological constituents of Epimedii Herba, pharmacokinetic studies have identified Icariin as a major bioactive pharmaceutical component [33], which exhibits a wide range of pharmacological activities [2,12,34,35,36]. The pharmacokinetic analysis of Icariin metabolism in rodents discovered the main metabolites of icariin: icaritin, icariside I, icariside II, and desmethylicaritin [37]. Although the exact metabolism of Icariin is not fully understood, it is now known that Icariin is metabolized in the body primarily in the liver through various pathways. Icariin is metabolized to several metabolites in the liver, which show a wide range of cardioprotective effects by inhibiting oxidative stress, reducing inflammation response, regulating cell apoptosis, and attenuating cellular senescence [38,39,40]. These cardiovascular protective characteristics make Icariin a candidate for treating MI.

Network pharmacology is an interdisciplinary field that seeks to understand the complex interactions between drugs, biological pathways, and disease mechanisms [17]. This is achieved through the use of network analysis and computational methods. The objective of network pharmacology is to identify the most effective combinations of drugs for treating various diseases [18,19]. This is accomplished by analyzing the interactions between drug targets and biological pathways. The result of this analysis can lead to the development of new drugs, improvements in existing treatments, and a deeper understanding of the underlying mechanisms of disease. In terms of methodology, network pharmacology utilizes complex data sets, including information on drug targets, biological pathways, and molecular interactions, to build models of drug–target interactions. These models can be used to identify new drug targets, predict potential side effects, and optimize drug delivery. The application of network pharmacology has the potential to greatly impact the field of drug discovery and development. Its interdisciplinary approach can contribute to a more comprehensive understanding of the relationships between drugs and the human body, leading to the advancement of treatments for various diseases [17]. Based on the network pharmacology strategies, our study revealed the potential targets of Icariin on MI, including *EGFR*, *AKT1*, *TP53*, *JUN*, *ESR1*, *PTGS2*, *TNF*, *RELA*, *HSP90AA1*, *BCL2L1*. The biological process and KEGG pathway enrichment analysis suggested that the protective effect of Icariin against MI might be associated with inhibited oxidative stress.

In MI, the lack of oxygen and nutrients delivered to the heart tissue leads to cellular damage and death, resulting in the formation of an infarct. Many pathological processes were involved in myocardial infarction, including platelet activation and aggregation, ischemia, inflammation, necrosis, and so forth. Importantly, ROS plays a key role in contributing to the development of many cardiovascular diseases. ROS are highly reactive molecules that are produced by cells as a byproduct of normal cellular metabolism and are also generated by various sources of oxidative stress, such as hypoxia, ischemia, and inflammation. In the development of MI, ROS contribute to the injury and death of heart muscle cells through multiple mechanisms. One of the key mechanisms by which ROS contribute to MI is through the oxidative modification of proteins and lipids, leading to cellular dysfunction and death. ROS can also induce the activation of various signaling pathways that contribute to the development of an inflammatory response, further exacerbating the injury to heart muscle cells. Our results indicated that the potential antioxidative effect of Icariin might benefit MI patients, probably from the polyphenol structure similar to other antioxidant properties. Zhao and colleagues [41] used a cell-free system to examine the anti-oxidant effects of Icariin, and they observed reduced DNA damage after Icariin treatment. Xia et al. [42] reported that Icariin could inhibit oxidative stress, inflammation, and apoptosis in chemotherapy-induced cardiotoxicity. The myocardial injury was significantly reduced via activating PI3K/Akt, inhibiting the MAPKs signaling pathway, and downregulating the secretion of inflammatory factors in H9c2 cells and mice models after Icariin treatment [42]. In addition, Icariin can inhibit ROS-induced JNK and p38 signaling pathways and thus protect cardiomyocytes from apoptosis [43]. Moreover, the anti-atherosclerosis effect of Icariin was also observed in ApoE^-/-^ mice [44,45], rats [46], and rabbit models [47].

TNF, tumor necrosis factor, is a pro-inflammatory cytokine that plays a crucial role in the pathological processes involved in cardiovascular diseases [48,49]. The secretion, release, and transformation of TNF is a mediator in the development and progress of MI. TNF is produced by various cell types, including immune cells, and is involved in the regulation of the immune response. It is reported that TNF contributes to the injury and death of heart muscle cells by multiple mechanisms. One of the key mechanisms by which TNF contributes to myocardial infarction is through the induction of oxidative stress. TNF can induce the production of reactive oxygen species (ROS), which are highly reactive molecules that contribute to the oxidative modification of proteins and lipids and the death of heart muscle cells. TNF can also induce the activation of various signaling pathways that contribute to the development of an inflammatory response, further exacerbating the injury to heart muscle cells.

Based on the high-cholesterol diet-induced atherosclerosis rat model, Hu et al. [46] reported that Icariin could inhibit atherosclerosis by reducing the circulating levels of TNF-α and IL-6 in a dose-dependent manner via p38/MAPK signaling pathway. Another study also revealed that Icariin could downregulate the mRNA levels of TNF-α, ICAM-1, IL-2, and IL-6, which attenuates myocardial inflammation [50]. This study identified the TNF as a hub gene in the Icariin treatment of MI. The molecular docking simulated the binding between Icariin and TNF protein. Last but not least, this study was not validated in a cell or animal model, which is a significant limitation. In the following studies, more external experiments should be performed to validate the results.

## 5. Conclusions

This study provides preliminary evidence for Icariin administration to treat MI. Further research is required to demonstrate the clinical benefit of Icariin on the development and progress of MI.

## Data Availability

The data used in this article were acquired from open-access databases, including PubChem, Swiss Target Prediction, STITCH, TTD, DrugBank, DisGeNET, and CTD databases.

## Supplementary Materials

The following supporting information can be downloaded at: https://www.mdpi.com/article/10.3390/medicina59030420/s1, Table S1. The myocardial infarction-related genes; Table S2. The Icariin target genes.

## References

[1] Mehta A., Mahtta D., Gulati M., Sperling L.S., Blumenthal R.S., Virani S.S. (2020). Cardiovascular Disease Prevention in Focus: Highlights from the 2019 American Heart Association Scientific Sessions. Curr. Atheroscler. Rep..

[2] Benjamin E.J., Muntner P., Alonso A., Bittencourt M.S., Callaway C.W., Carson A.P., Chamberlain A.M., Chang A.R., Cheng S., Das S.R. (2019). American Heart Association Council on Epidemiology and Prevention Statistics Committee and Stroke Statistics Subcommittee. Heart Disease and Stroke Statistics-2019 Update: A Report From the American Heart Association. Circulation.

[3] Yu B., Akushevich I., Yashkin A.P., Kravchenko J. (2021). Epidemiology of Geographic Disparities of Myocardial Infarction Among Older Adults in the United States: Analysis of 2000-2017 Medicare Data. Front. Cardiovasc. Med..

[4] Benjamin E.J., Virani S.S., Callaway C.W., Chamberlain A.M., Chang A.R., Cheng S., Chiuve S.E., Cushman M., Delling F.N., Deo R. (2018). Heart Disease and Stroke Statistics-2018 Update: A Report From the American Heart Association. Circulation.

[5] Ambrose J.A., Singh M. (2015). Pathophysiology of coronary artery disease leading to acute coronary syndromes. F1000Prime Rep..

[6] Liu Y., Chen C., Wang X., Sun Y., Zhang J., Chen J., Shi Y. (2022). An Epigenetic Role of Mitochondria in Cancer. Cells.

[7] Chen K., Zhang J., Beeraka N.M., Tang C., Babayeva Y.V., Sinelnikov M.Y., Zhang X., Zhang J., Liu J., Reshetov I.V. (2022). Advances in the Prevention and Treatment of Obesity-Driven Effects in Breast Cancers. Front. Oncol..

[8] Chen K., Lu P., Beeraka N.M., Sukocheva O.A., Madhunapantula S.V., Liu J., Sinelnikov M.Y., Nikolenko V.N., Bulygin K.V., Mikhaleva L.M. (2022). Mitochondrial mutations and mitoepigenetics: Focus on regulation of oxidative stress-induced responses in breast cancers. Semin. Cancer Biol..

[9] Shekhar S., Liu Y., Wang S., Zhang H., Fang X., Zhang J., Fan L., Zheng B., Roman R.J., Wang Z. (2021). Novel Mechanistic Insights and Potential Therapeutic Impact of TRPC6 in Neurovascular Coupling and Ischemic Stroke. Int. J. Mol. Sci..

[10] Ye L.C., Chen J.M. (2001). [Advances in study on pharmacological effects of Epimedium].. Zhongguo Zhong Yao Za Zhi.

[11] Fang J., Zhang Y. (2017). Icariin, an Anti-atherosclerotic Drug from Chinese Medicinal Herb Horny Goat Weed. Front. Pharm..

[12] Xu H.B., Huang Z.Q. (2007). Icariin enhances endothelial nitric-oxide synthase expression on human endothelial cells in vitro. Vasc. Pharm..

[13] Wu B., Feng J.Y., Yu L.M., Wang Y.C., Chen Y.Q., Wei Y., Han J.S., Feng X., Zhang Y., Di S.Y. (2018). Icariin protects cardiomyocytes against ischaemia/reperfusion injury by attenuating sirtuin 1-dependent mitochondrial oxidative damage. Br. J. Pharm..

[14] Song Y.H., Cai H., Zhao Z.M., Chang W.J., Gu N., Cao S.P., Wu M.L. (2016). Icariin attenuated oxidative stress induced-cardiac apoptosis by mitochondria protection and ERK activation. Biomed. Pharm..

[15] Zhai M., He L., Ju X., Shao L., Li G., Zhang Y., Liu Y., Zhao H. (2015). Icariin Acts as a Potential Agent for Preventing Cardiac Ischemia/Reperfusion Injury. Cell Biochem. Biophys..

[16] Shi Y., Yan W., Lin Q., Wang W. (2018). Icariin influences cardiac remodeling following myocardial infarction by regulating the CD147/MMP-9 pathway. J. Int. Med. Res..

[17] Liu X.J., Lv Y.F., Cui W.Z., Li Y., Liu Y., Xue Y.T., Dong F. (2021). Icariin inhibits hypoxia/reoxygenation-induced ferroptosis of cardiomyocytes via regulation of the Nrf2/HO-1 signaling pathway. FEBS Open Bio..

[18] Noor F., Tahir Ul Qamar M., Ashfaq U.A., Albutti A., Alwashmi A.S.S., Aljasir M.A. (2022). Network Pharmacology Approach for Medicinal Plants: Review and Assessment. Pharmaceuticals.

[19] Hopkins A.L. (2008). Network pharmacology: The next paradigm in drug discovery. Nat. Chem. Biol..

[20] Kibble M., Saarinen N., Tang J., Wennerberg K., Makela S., Aittokallio T. (2015). Network pharmacology applications to map the unexplored target space and therapeutic potential of natural products. Nat. Prod. Rep..

[21] Xiong Y., Yang Y., Xiong W., Yao Y., Wu H., Zhang M. (2019). Network pharmacology-based research on the active component and mechanism of the antihepatoma effect of *Rubia cordifolia* L. J. Cell Biochem..

[22] Shi M.J., Yan X.L., Dong B.S., Yang W.N., Su S.B., Zhang H. (2020). A network pharmacology approach to investigating the mechanism of Tanshinone IIA for the treatment of liver fibrosis. J. Ethnopharmacol..

[23] Daina A., Michielin O., Zoete V. (2019). SwissTargetPrediction: Updated data and new features for efficient prediction of protein targets of small molecules. Nucleic Acids Res..

[24] Szklarczyk D., Santos A., von Mering C., Jensen L.J., Bork P., Kuhn M. (2016). STITCH 5: Augmenting protein-chemical interaction networks with tissue and affinity data. Nucleic Acids Res..

[25] Wang Y., Zhang S., Li F., Zhou Y., Zhang Y., Wang Z., Zhang R., Zhu J., Ren Y., Tan Y. (2020). Therapeutic target database 2020: Enriched resource for facilitating research and early development of targeted therapeutics. Nucleic Acids Res..

[26] Pinero J., Ramirez-Anguita J.M., Sauch-Pitarch J., Ronzano F., Centeno E., Sanz F., Furlong L.I. (2020). The DisGeNET knowledge platform for disease genomics: 2019 update. Nucleic Acids Res..

[27] Davis A.P., Grondin C.J., Johnson R.J., Sciaky D., McMorran R., Wiegers J., Wiegers T.C., Mattingly C.J. (2019). The Comparative Toxicogenomics Database: Update 2019. Nucleic Acids Res..

[28] UniProt Consortium (2019). UniProt: A worldwide hub of protein knowledge. Nucleic Acids Res..

[29] Szklarczyk D., Gable A.L., Lyon D., Junge A., Wyder S., Huerta-Cepas J., Simonovic M., Doncheva N.T., Morris J.H., Bork P. (2019). STRING v11: Protein-protein association networks with increased coverage, supporting functional discovery in genome-wide experimental datasets. Nucleic Acids Res..

[30] Shannon P., Markiel A., Ozier O., Baliga N.S., Wang J.T., Ramage D., Amin N., Schwikowski B., Ideker T. (2003). Cytoscape: A software environment for integrated models of biomolecular interaction networks. Genome Res..

[31] Meng X.Y., Zhang H.X., Mezei M., Cui M. (2011). Molecular docking: A powerful approach for structure-based drug discovery. Curr. Comput. Aided Drug Des..

[32] Benjamin E.J., Muntner P., Alonso A., Bittencourt M.S., Callaway C.W., Carson A.P., Chamberlain A.M., Chang A.R., Cheng S., Das S.R. (2019). Heart Disease and Stroke Statistics-2019 Update: A Report From the American Heart Association. Circulation.

[33] Liu J., Ye H., Lou Y. (2005). Determination of rat urinary metabolites of icariin in vivo and estrogenic activities of its metabolites on MCF-7 cells. Pharmazie.

[34] Wang Z., Wang D., Yang D., Zhen W., Zhang J., Peng S. (2018). The effect of icariin on bone metabolism and its potential clinical application. Osteoporos. Int..

[35] Jin J., Wang H., Hua X., Chen D., Huang C., Chen Z. (2019). An outline for the pharmacological effect of icariin in the nervous system. Eur. J. Pharm..

[36] Tan H.L., Chan K.G., Pusparajah P., Saokaew S., Duangjai A., Lee L.H., Goh B.H. (2016). Anti-Cancer Properties of the Naturally Occurring Aphrodisiacs: Icariin and Its Derivatives. Front. Pharm..

[37] Bi Z., Zhang W., Yan X. (2022). Anti-inflammatory and immunoregulatory effects of icariin and icaritin. Biomed. Pharm..

[38] Sharma S., Khan V., Dhyani N., Najmi A.K., Haque S.E. (2020). Icariin attenuates isoproterenol-induced cardiac toxicity in Wistar rats via modulating cGMP level and NF-kappaB signaling cascade. Hum. Exp. Toxicol..

[39] Mi B., Liu J., Liu G., Zhou W., Liu Y., Hu L., Xiong L., Ye S., Wu Y. (2018). Icariin promotes wound healing by enhancing the migration and proliferation of keratinocytes via the AKT and ERK signaling pathway. Int. J. Mol. Med..

[40] Mi B., Wang J., Liu Y., Liu J., Hu L., Panayi A.C., Liu G., Zhou W. (2018). Icariin Activates Autophagy via Down-Regulation of the NF-kappaB Signaling-Mediated Apoptosis in Chondrocytes. Front. Pharm..

[41] Zhao F., Tang Y.Z., Liu Z.Q. (2007). Protective effect of icariin on DNA against radical-induced oxidative damage. J. Pharm. Pharm..

[42] Xia J., Hu J.N., Zhang R.B., Liu W., Zhang H., Wang Z., Jiang S., Wang Y.P., Li W. (2022). Icariin exhibits protective effects on cisplatin-induced cardiotoxicity via ROS-mediated oxidative stress injury in vivo and in vitro. Phytomedicine.

[43] Zhou H., Yuan Y., Liu Y., Deng W., Zong J., Bian Z.Y., Dai J., Tang Q.Z. (2014). Icariin attenuates angiotensin II-induced hypertrophy and apoptosis in H9c2 cardiomyocytes by inhibiting reactive oxygen species-dependent JNK and p38 pathways. Exp. Med..

[44] Xiao H.B., Liu Z.K., Lu X.Y., Deng C.N., Luo Z.F. (2015). Icariin regulates PRMT/ADMA/DDAH pathway to improve endothelial function. Pharm. Rep..

[45] Wang Y., Wang Y.S., Song S.L., Liang H., Ji A.G. (2016). Icariin inhibits atherosclerosis progress in Apoe null mice by downregulating CX3CR1 in macrophage. Biochem. Biophys. Res. Commun..

[46] Hu Y., Sun B., Liu K., Yan M., Zhang Y., Miao C., Ren L. (2016). Icariin Attenuates High-cholesterol Diet Induced Atherosclerosis in Rats by Inhibition of Inflammatory Response and p38 MAPK Signaling Pathway. Inflammation.

[47] Zhang W.P., Bai X.J., Zheng X.P., Xie X.L., Yuan Z.Y. (2013). Icariin attenuates the enhanced prothrombotic state in atherosclerotic rabbits independently of its lipid-lowering effects. Planta Med..

[48] Tian M., Yuan Y.C., Li J.Y., Gionfriddo M.R., Huang R.C. (2015). Tumor necrosis factor-alpha and its role as a mediator in myocardial infarction: A brief review. Chronic Dis. Transl. Med..

[49] Hamid T., Gu Y., Ortines R.V., Bhattacharya C., Wang G., Xuan Y.T., Prabhu S.D. (2009). Divergent tumor necrosis factor receptor-related remodeling responses in heart failure: Role of nuclear factor-kappaB and inflammatory activation. Circulation.

[50] Chen Y., Sun T., Wu J., Kalionis B., Zhang C., Yuan D., Huang J., Cai W., Fang H., Xia S. (2015). Icariin intervenes in cardiac inflammaging through upregulation of SIRT6 enzyme activity and inhibition of the NF-kappa B pathway. Biomed. Res. Int..

